# Supplementary figures and images for: Nucleocapsid mutations in SARS-CoV-2 augment replication and pathogenesis
Source: PLoS Pathog. 2022 Jun 21;18(6):e1010627. doi: 10.1371/journal.ppat.1010627 (PMC9275689; doi:10.1371/journal.ppat.1010627)

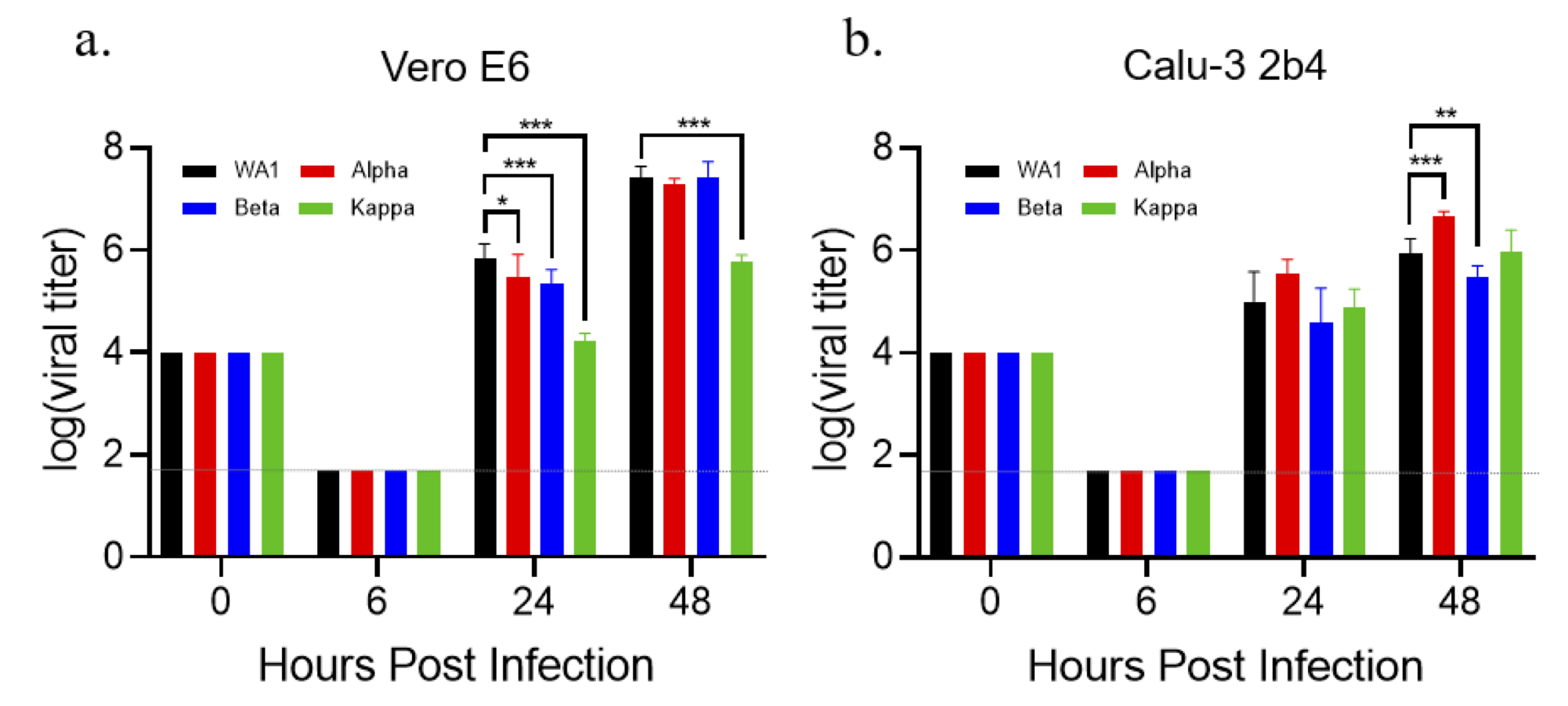

Supplement: S1 Fig — Viral titer from Vero E6 (A) or Calu-3 2b4 cells (B) inoculated with SARS-CoV-2 WA-1 (black) or the alpha (red), beta (blue) or kappa (green) variants at a MOI of 0.01. Graphed data represent the mean ± s.d. Statistical significance was determined by two-tailed student’s T-test with p≤0.05 (*), p≤0.01 (**), and p≤ 0.001 (***). Grey dotted lines are equal to LOD. (TIFF) [file ppat.1010627.s001.tiff]

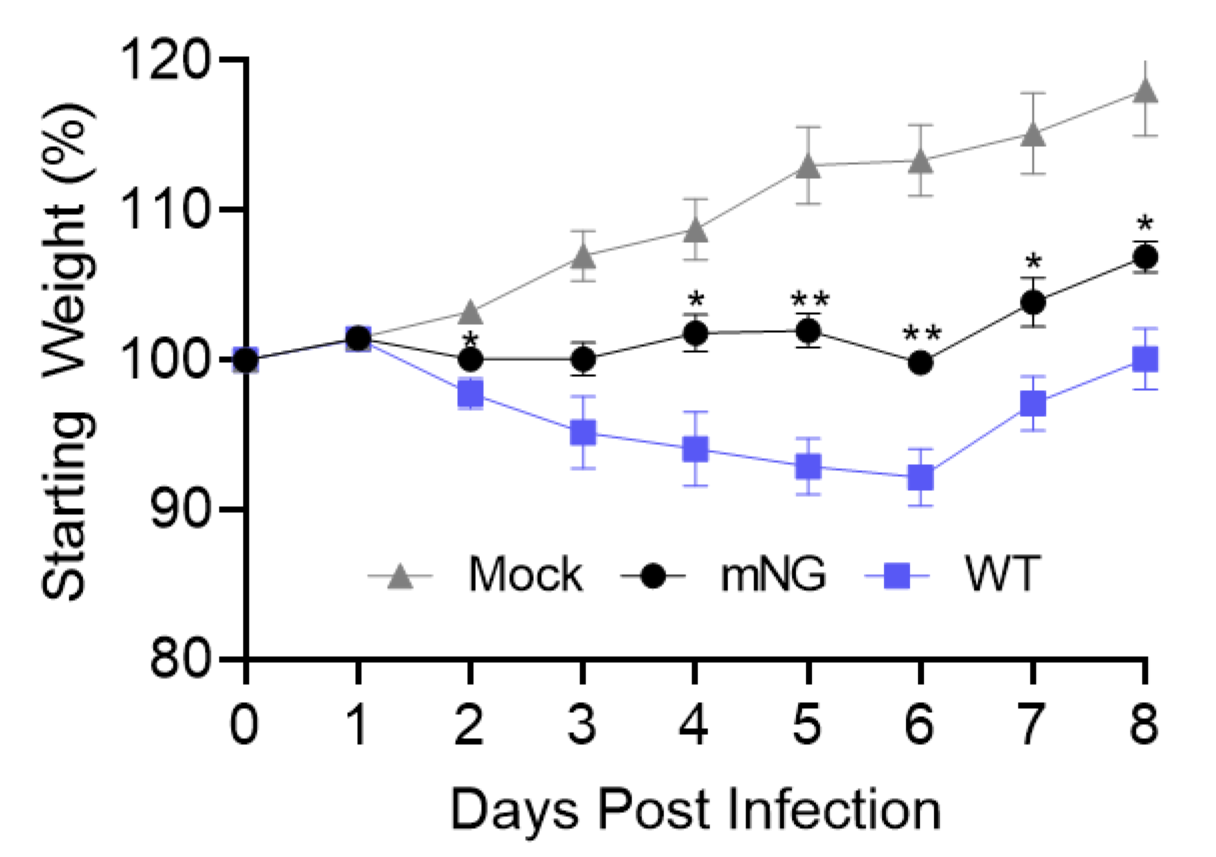

Supplement: S2 Fig — Three- to four-week-old Golden Syrian hamsters were intranasally inoculated with PBS alone (gray) or 104 PFU of WA-1 SARS-CoV-2 (blue) or mNG SARS-CoV-2 (black). Graphed data represent the mean weight loss ± s.e.m (n≥5). Statistical significance between WT and mNG determined by two-tailed students T-test with p≤0.05 (*) and p≤0.01 (**). (TIFF) [file ppat.1010627.s002.tiff]

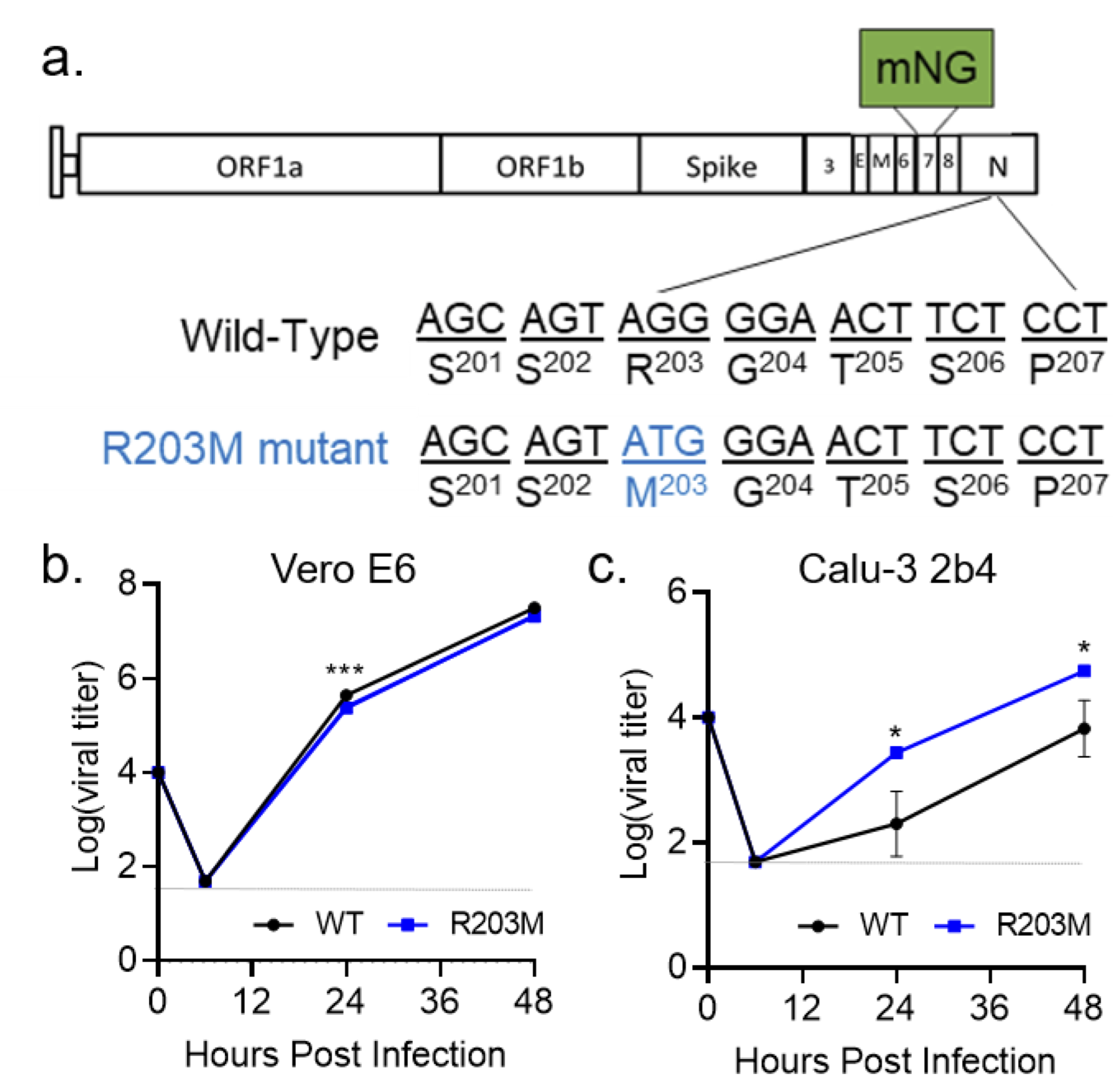

Supplement: S3 Fig — (A) Schematic of the SARS-CoV-2 genome, showing the creation of the R203M mutation and the replacement of ORF7 with the mNG reporter protein. (B-C) Viral titers from Vero E6 (B) or Calu-3 2b4 (C) infected with WT or R203M SARS-CoV-2 at an MOI of 0.01. Graphed data represent mean ± s.d. (n = 3). Statistical significance was determined by two-tailed student’s T-test with p≤0.05 (*) and p≤ 0.001 (***). Grey dotted lines are equal to LOD. (TIFF) [file ppat.1010627.s003.tiff]

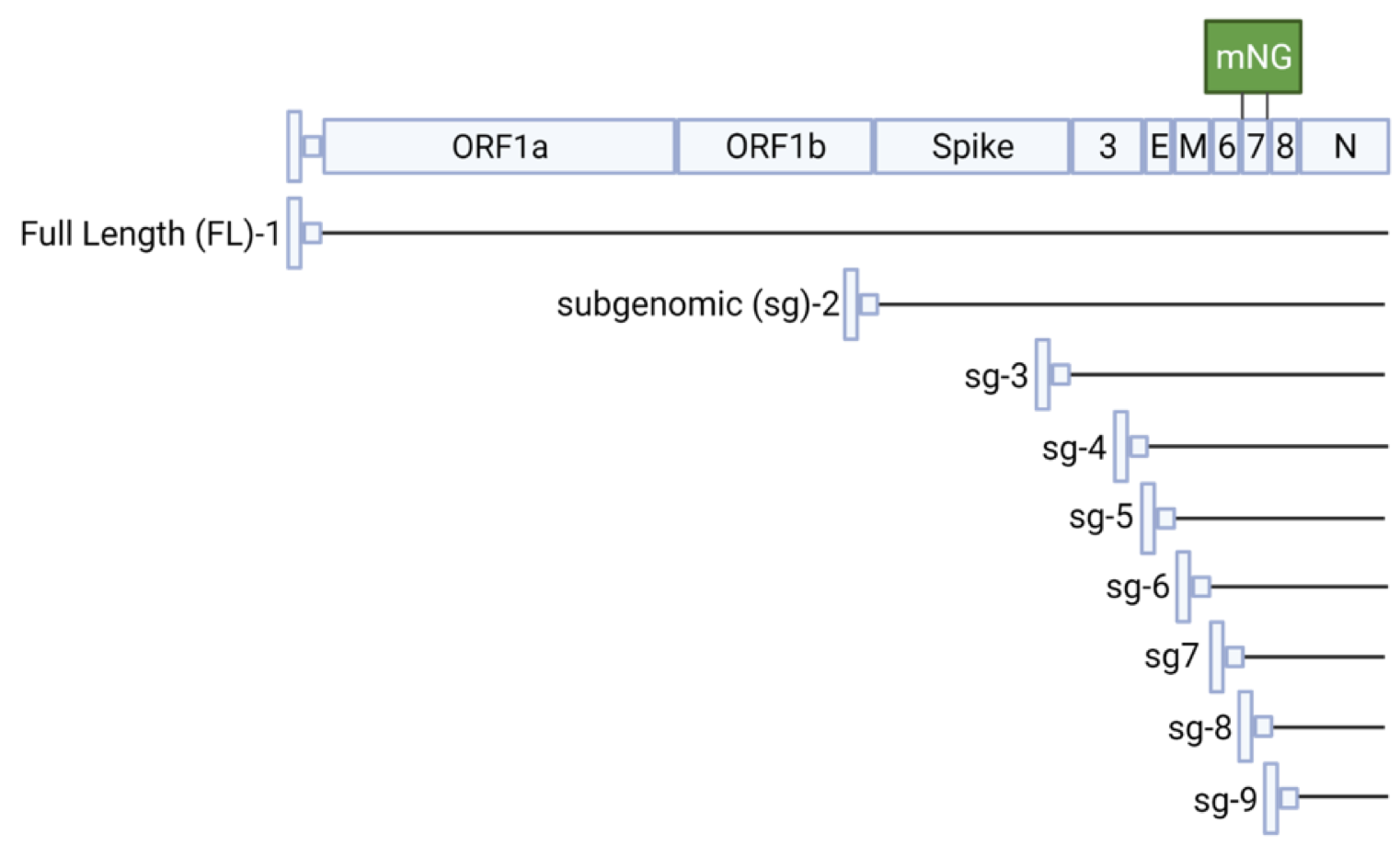

Supplement: S4 Fig — Illustration of full length (FL) and subgenomic (sg) RNAs produced during SARS-CoV-2 infection. (TIFF) [file ppat.1010627.s004.tiff]

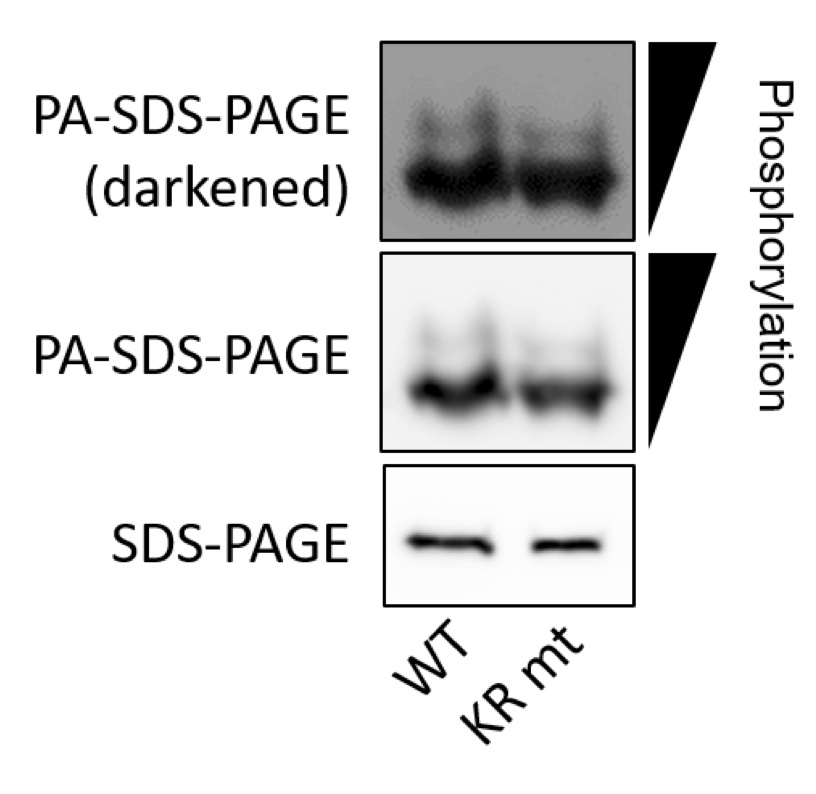

Supplement: S5 Fig — Calu-3 2b4 cells were infected at an MOI of 0.01 with WT or KR mt SARS-CoV-2. Forty-eight hours post infection, viral supernatants were taken. Virions were then purified from supernatants by ultracentrifugation on a 20% sucrose cushion, inactivated, and N levels analyzed by both phospho-affinity and standard SDS-Page. Results are representative of two independent experiments. (TIFF) [file ppat.1010627.s005.tiff]

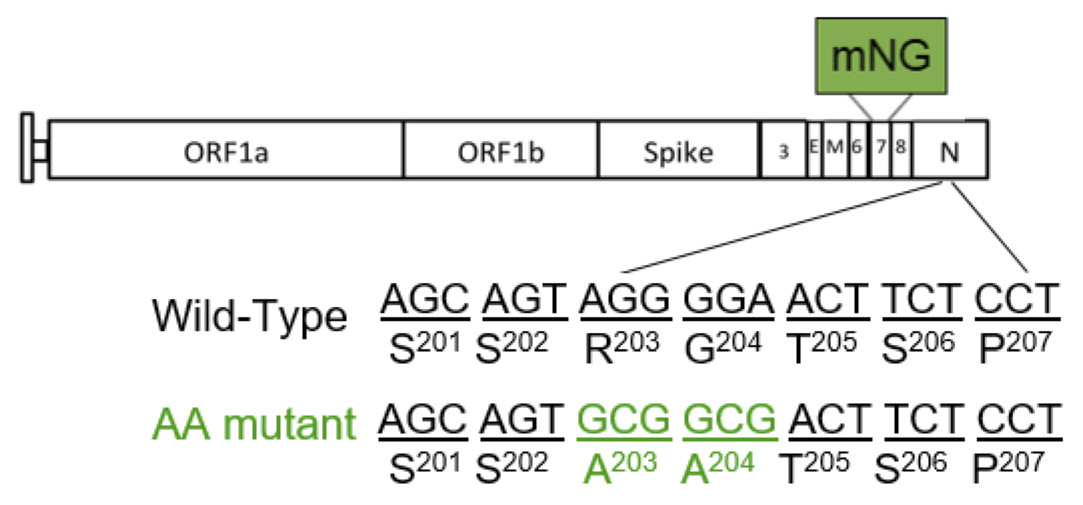

Supplement: S6 Fig — Schematic shows the creation of the AA mutation within the SARS-CoV-2 genome and the replacement of ORF7 with the mNeonGreen reporter. (TIFF) [file ppat.1010627.s006.tiff]

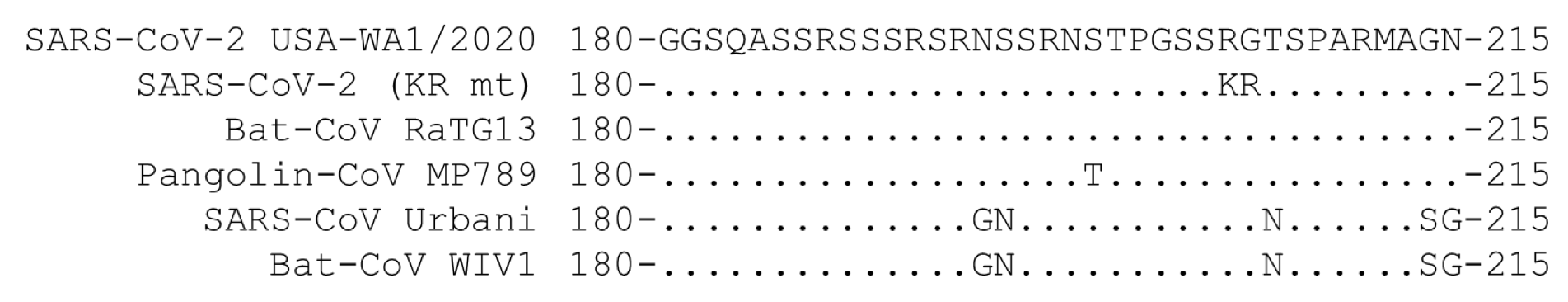

Supplement: S7 Fig — Amino acid sequence alignment of SARS-CoV-2 residues 180–215. Genbank accession numbers for each virus are SARS-CoV-2 USA-WA1/2020 (MN985325.1), Bat-CoV RaTG13 (MN996532.2), Pangolin-CoV MP789 (MT121216.1), SARS-CoV Urbani (AY278741.1), and Bat-CoV WIV1 (KF367457.1). (TIFF) [file ppat.1010627.s007.tiff]

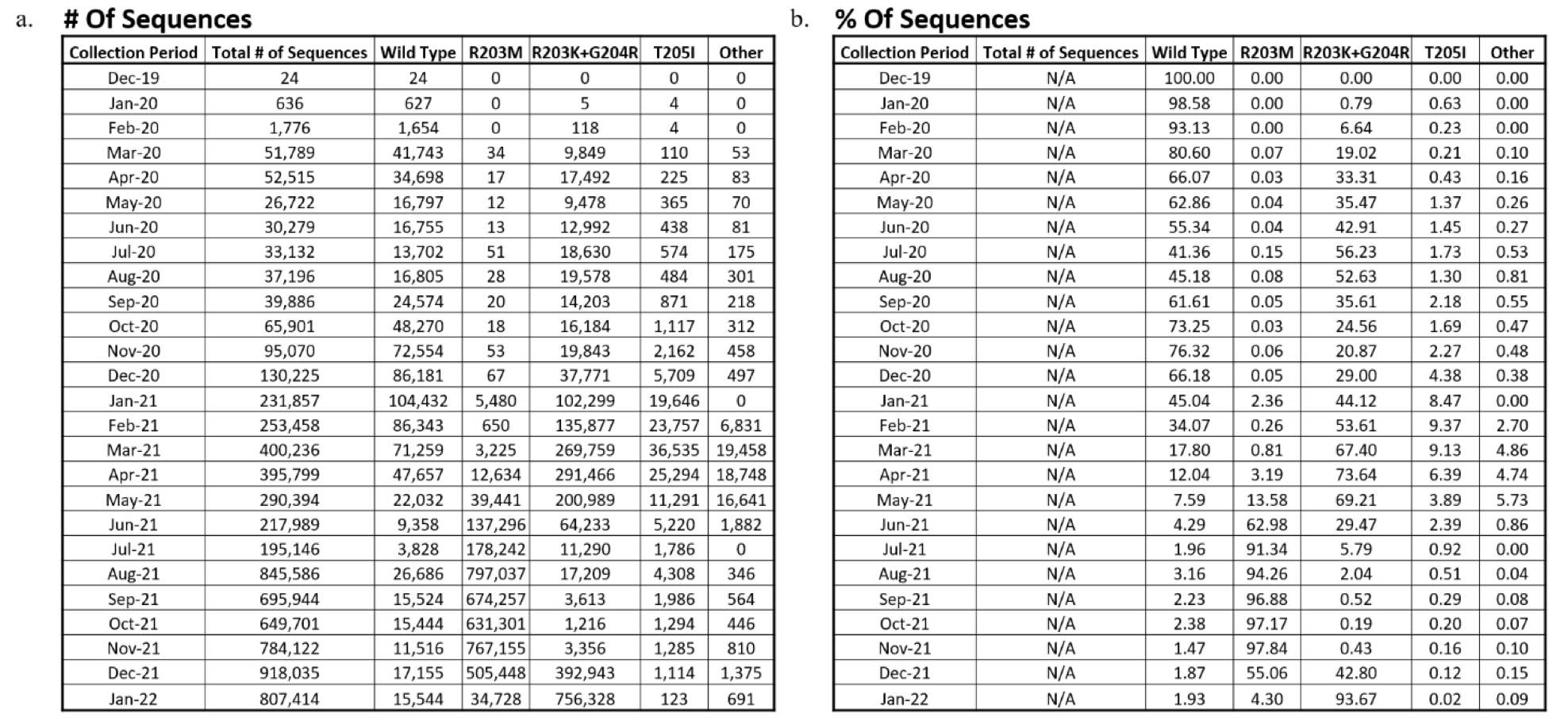

Supplement: S1 Table — (A-B) Frequency of WT, R203M, R203K+G204R, T205I, or all other genotypes binned by month of collection, represented as the raw totals (A) or as a percentage of total sequences in a given month (B). (TIFF) [file ppat.1010627.s008.tiff]
